# Diversity and Distribution of Freshwater Amphipod Species in Switzerland (Crustacea: Amphipoda)

**DOI:** 10.1371/journal.pone.0110328

**Published:** 2014-10-29

**Authors:** Florian Altermatt, Roman Alther, Cene Fišer, Jukka Jokela, Marjeta Konec, Daniel Küry, Elvira Mächler, Pascal Stucki, Anja Marie Westram

**Affiliations:** 1 Department of Aquatic Ecology, Eawag: Swiss Federal Institute of Aquatic Science and Technology, Dübendorf, Switzerland; 2 Department of Environmental Systems Science, ETH Zentrum, Zürich, Switzerland; 3 Institute of Evolutionary Biology and Environmental Studies, University of Zurich, Zürich, Switzerland; 4 Department of Biology, Biotechnical Faculty, University of Ljubljana, Ljubljana, Slovenia; 5 Life Science AG, Basel, Switzerland; 6 Aquabug, Neuchâtel, Switzerland; 7 Animal and Plant Sciences, University of Sheffield, Western Bank, Sheffield, United Kingdom; Consiglio Nazionale delle Ricerche (CNR), Italy

## Abstract

Amphipods are key organisms in many freshwater systems and contribute substantially to the diversity and functioning of macroinvertebrate communities. Furthermore, they are commonly used as bioindicators and for ecotoxicological tests. For many areas, however, diversity and distribution of amphipods is inadequately known, which limits their use in ecological and ecotoxicological studies and handicaps conservation initiatives. We studied the diversity and distribution of amphipods in Switzerland (Central Europe), covering four major drainage basins, an altitudinal gradient of>2,500 m, and various habitats (rivers, streams, lakes and groundwater). We provide the first provisional checklist and detailed information on the distribution and diversity of all amphipod species from Switzerland. In total, we found 29 amphipod species. This includes 16 native and 13 non-native species, one of the latter (*Orchestia cavimana*) reported here for the first time for Switzerland. The diversity is compared to neighboring countries. We specifically discuss species of the genus *Niphargus*, which are often receiving less attention. We also found evidence of an even higher level of hidden diversity, and the potential occurrence of further cryptic species. This diversity reflects the biogeographic past of Switzerland, and suggests that amphipods are ideally suited to address questions on endemism and adaptive radiations, post-glaciation re-colonization and invasion dynamics as well as biodiversity-ecosystem functioning relationships in aquatic systems.

## Introduction

Understanding the diversity and distribution of organisms is a fundamental goal of ecology, and a prerequisite for using species in monitoring programs or as bioindicators. This is especially relevant for freshwater systems, which are highly diverse, but also highly threatened [1], [2], and for which the occurrence of characteristic diversity patterns is postulated [3]. While the diversity and distribution of freshwater vertebrates, such as birds, fish or mammals, is generally well-known, knowledge on invertebrates is often more limited.

**Figure 1:**
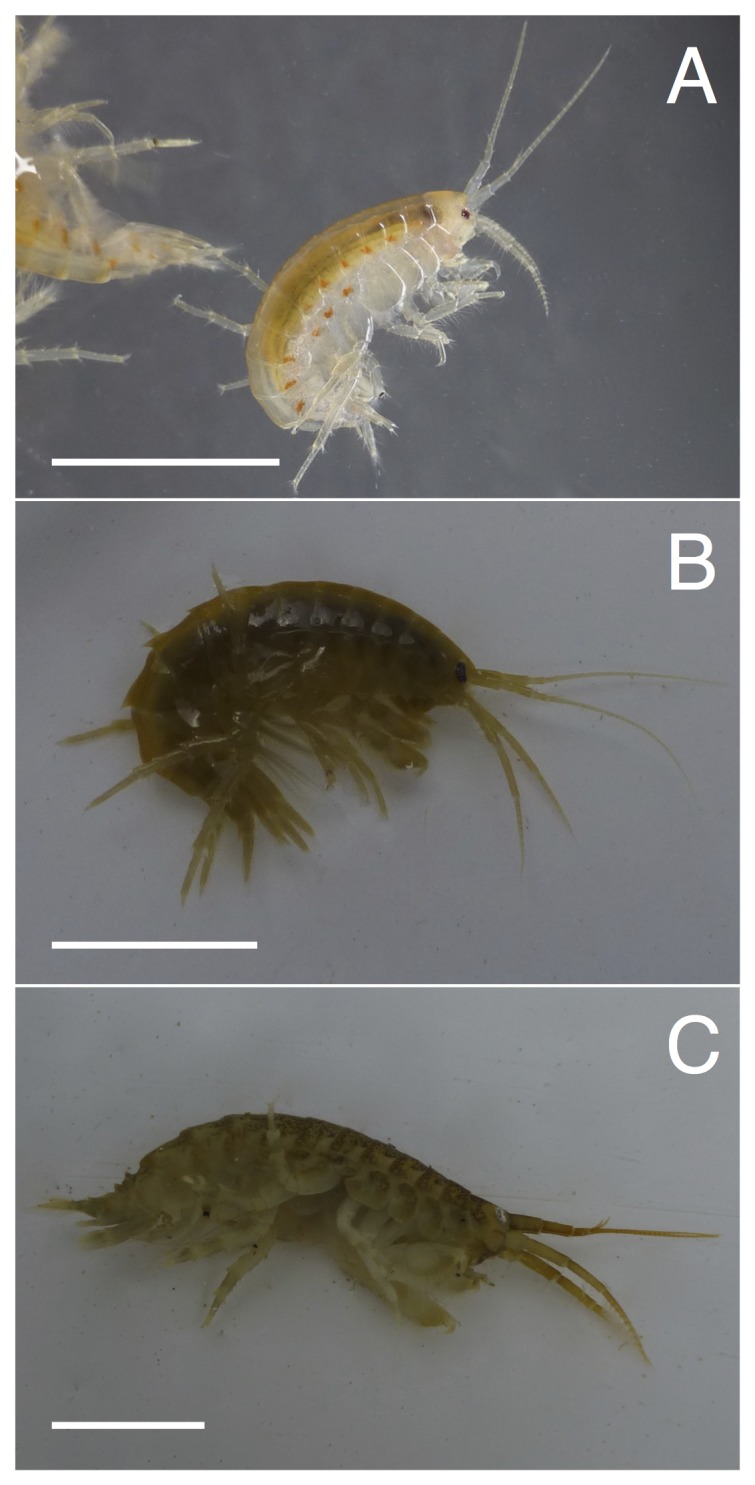
Morphological diversity within the order of Amphipoda. Three (of the in total 29) different species/species complexes known from Switzerland are shown: A) *Gammarus fossarum* complex, B) *Gammarus roeseli* and C) *Dikerogammarus villosus*. *G. fossarum* is native to Switzerland, *G. roeseli* is a non-native species that arrived in Switzerland around 1850, and *D. villosus* is a non-native species that arrived in Switzerland in the late 1990s. The scale bar is equivalent to 1 cm and gives approximate size differences between the species. The diverse color patterns visible in these pictures of living animals are completely lost in specimens preserved in alcohol. All pictures by Florian Altermatt.

Amphipods (class Crustacea, order Amphipoda; Fig. 1) are an important and diverse group of macroinvertebrates [4], [5], many of which inhabit freshwater environments including epibenthic, benthic and subterranean habitats. Worldwide, about 2,000 species of freshwater amphipods are known, with 70% of these species found in the Palaearctic [4]. Even though they can contribute substantially to the diversity and biomass of aquatic communities, detailed knowledge on the distribution and community composition of freshwater amphipods is lacking for many regions. While endemic species of lake Baikal or karst regions of south-eastern Europe (e.g., Italy or Slovenia) have been studied intensively (e.g., [4], [6]–[8]), conclusive information on the distribution and diversity of amphipods is lacking for some alpine regions, especially for Switzerland (Table 1). This is unfortunate, as the European Alps represent a diversity hotspot for many groups of aquatic species. Multiple cycles of glaciation and re-colonizations from refugia and a complex geology have resulted in a mosaic of species' distributions (e.g., [9], [10]). The Swiss Alps form major continental drainage systems (origin or tributaries to the rivers Rhine, Rhone, Danube, and Po), and thus have been and are open for colonization from biogeographically different regions. This has led to a different faunal composition north and south of the Swiss Alps for many groups of organisms, including frequent adaptive radiations and high degrees of endemism, for example in whitefish (*Coregonus* sp. [10]), or in may- and stoneflies [11], [12].

**Table 1:** Overview of the hitherto published diversity of Amphipoda in Switzerland, neighboring countries of Switzerland (Austria, Germany, Italy, France) as well as Slovenia.

country	Nr of families	Nr of genera	Nr of species
Austria	3/–	6/–	16/–
France	8/–	16/–	67/–
Germany	5/8	12/17	36/48
Italy	8/11	16/18	68/119
Slovenia	4/9	8/11	38/55
Switzerland	2/–	4/–	12/–

The latter is especially well-studied and and therefore given for comparison. For each country, diversity at the family, genus and species level is given. We first give the number of taxa at each level from Fauna Europaea [77] and after the diagonal slash from other overview publications screened (when available, a list of these publications is given in the Method section). In case of missing or incomplete data at the country level (e.g., no publication considering all species within the order Amphipoda), a dash "–" is given.

However, recent anthropogenic changes in the connectivity of river systems and loss of dispersal limitation also resulted in a higher inflow of non-native invertebrate species [13]. Amphipods are not only among the most successful but also among the most common invasive invertebrate species [14], [15], capable of shifting whole communities of aquatic macroinvertebrates. Invasive species are currently changing the diversity and composition of amphipod communities in many countries, including Switzerland.

In parallel of a high ecological significance, amphipods are receiving an increasing interest in eco-toxicological and environmental biomonitoring (e.g., [16]–[18]). However, this work has been made difficult by major gaps in the basic distribution data and fundamental difficulties in morphological identification of amphipods: the relevant morphology-based taxonomic keys on amphipods are challenged by a very high intra- and inter-population variation in morphology (e.g., [15], [19]–[21]). As a result, detailed information on amphipods is lacking from Switzerland (Table 1). For other European areas/countries, presence-absence checklists or large-scale distribution data are available, while more detailed distribution data are usually only available for a subset of amphipod species (e.g., [7], [15], [22]–[24]). Subsequently, in many applied studies, correct species-level identification of amphipods is not done. This is a serious problem because different species may be inadvertently compared in ecotoxicological tests [25], or the presence and potential decline of species at a site is unrecognized, as only presence/absence of amphipods as a whole group is recorded [17].

Here, we provide the first provisional checklist and detailed information on the distribution and diversity of all amphipod species found in Switzerland to date. We include data from standardized federal and cantonal monitoring programs, literature, as well as from our own extensive fieldwork. Our database consists of>150,000 individuals collected at about 2,500 sites. Individuals were identified based on morphological and molecular methods, and include species from lakes, rivers, streams, and groundwater. We provide distribution maps and information on the altitudinal distribution of all native and non-native amphipod species known from Switzerland and compare the diversity to neighboring countries. Furthermore, we analyze community composition and co-occurrence of species, and identify diversity hotspots and invasion pathways.

## Material and Methods

### Study area

Our study area is Switzerland, covering an area of 41,285 km^2^. Switzerland contains the origin or important tributaries of four major alpine drainage systems (Rhine, Rhone, Inn/Danube, Ticino/Po, covering 71%, 20%, 5%, and 3.5% respectively of the country), which drain into the North Sea, the Mediterranean Sea, the Black Sea and the Adriatic Sea, respectively. Thereby, Switzerland reflects the diversity and biogeography of European headwaters. The country exhibits a large altitudinal range from 193 to 4634 m a.s.l. and covers a diversity of geological substrates, including karst, granite and alluvial sediments. A temperate climate and medium to high level of precipitation result in a large number of freshwater habitats.

### Data sources and sampling methods

**Figure 2:**
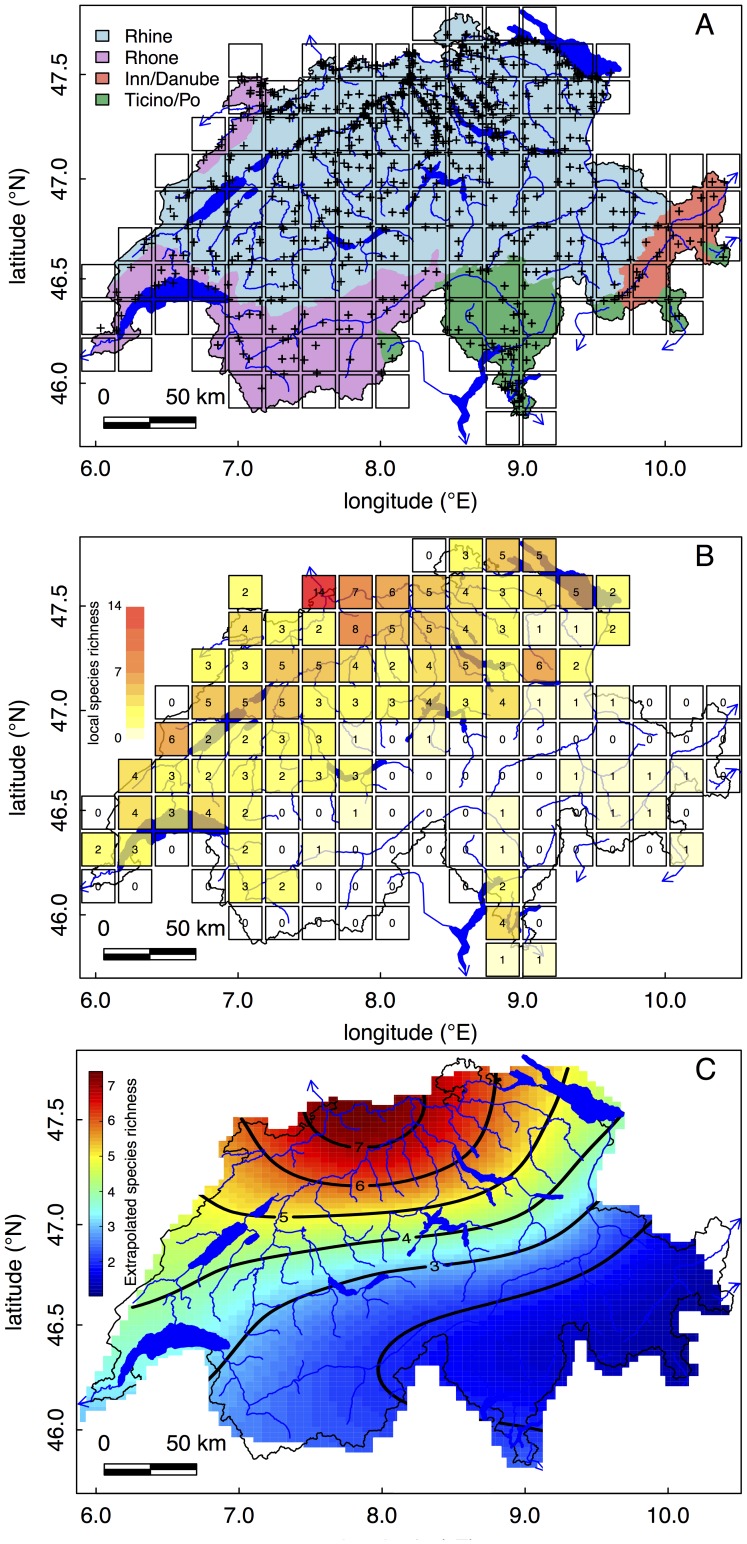
Sampling locations and diversity pattern of amphipods in Switzerland. A) Map of Switzerland showing all sampling sites included in our study (crosses). The four drainage basins (Rhine, Rhone, Inn/Danube and Ticino/Po) are given in different colors, and the major river and lakes are given in blue. The grid of the 20×20 km squares was used to calculate diversity patterns in panel B. B) Diversity of amphipods in 20×20 km squares covering all of Switzerland. Local species richness in each square is given as color gradient and a number. C) Interpolated fits of local amphipod species richness using a thin plate spline surface to irregularly spaced data.

We compiled a database containing amphipod records from literature references, museum collections, governmental monitoring programs, as well as records from our own extensive fieldwork. First, we screened all available literature on reliable amphipod records from Switzerland. This not only included published studies but also many unpublished reports conducted by federal or cantonal agencies (“grey literature”). Literature was acquired by a Web of Science search with “amphipod” and “Switzerland” as key words, complemented by a survey targeting aquatic ecologists, consultancy companies and governmental agencies in Switzerland. In total, over 30 references were evaluated and data thereof included [23], [26]–[55]. We only used literature records on amphipods when the identification and data source was traceable. Second, we screened museum collections for species for which we had only few records (especially Niphargidae). We screened the collections of the National History Museum in Basel, and the private collections of Aquabug (Neuchâtel) and LifeScience AG (Basel). Third, we identified all amphipod samples collected in the Biodiversity Monitoring Program of Switzerland (BDM, [12]). In this program, all macroinvertebrates are sampled based on highly standardized methods at over 500 randomly selected sites across the Swiss river and stream network since 2009. Finally, we conducted our own extensive fieldwork at>200 sites across Switzerland, targeting areas that were underrepresented by the other data-sources (e.g., Southern Switzerland/Ticino, Alpine Rhine valley, tributaries of Lake Constance, Jura mountains, and alpine valleys and alpine lakes). To access these sites and to do the sampling, no specific permission was required, as none of the sites were in protected areas and did not involve endangered or protected species. Field sampling was predominantly conducted by standardized kicknet sampling [17], following the protocols used in the BDM. Besides these standardized samplings, we also collected individuals by specifically targeting known microhabitats of amphipods, such as wells and groundwater systems, lakeshores and streams. All collected individuals were preserved in 70% ethanol. All data sources (except [23], [26]) looked at all amphipod species at the sampling sites, thereby not creating biases with respect to species groups identified. Sampling efforts were not completely evenly distributed across Switzerland (except for the BDM data), and some habitat types (deep lakes, natural springs/groundwater) are underrepresented. We give information on sampling intensity (Fig. 2A), thereby also identifying “white spots” with respect to sampling efforts.

We built a database containing information on the precise geographic location, elevation, habitat type, and identification method of all amphipod individuals considered. Individuals that could not be identified to the species level with neither morphological (e.g., juveniles or damaged specimens) nor molecular methods were excluded from all analyses. The database will be integrated into the Swiss Biological Records Center (www.CSCF.ch) to become publicly available.

### Morphological identification

We aimed at identifying all individuals to the most precise and commonly accepted taxonomic level. Using standard literature [15], [19]–[21], [56], we in a first step identified individuals to the species level based on morphological characters, using a stereomicroscope at 20- to 100-fold magnification. For all individuals of the genus *Niphargus*, morphological analyses were made using the original description of the species. Morphologically delimited species may still contain cryptic species, and we in a second step included genetic data for species identification for the *Gammarus fossarum* species complex and the genus *Niphargus*.

### Molecular identification of *Gammarus fossarum* species complex

*Gammarus fossarum* is known to be a species complex, containing at least three species (type A, B and C) in Switzerland, which cannot be told apart based on morphological characters only [22], [23], [26], [57]-[59]. We identified *G. fossarum* from as many sites as possible using previously established microsatellite and SNP markers for species identification. In total, we extracted DNA from about 4,500 individuals of the *G. fossarum*-complex, either extracting DNA from whole individuals or from pereopods, and analyzed ten microsatellite markers using the identical method as described in Westram et al. [59]. The occurrence of specific allelic combinations in these microsatellite markers is diagnostic for each of the three cryptic species, and corresponds to both species-specific SNP as well as COI sequences (for details see [23], [26], [59]). The microsatellite markers diagnostic for type A is gf27 polymorphic with alleles>200 bp (but ≠205), for type B the marker is monomorphic at 205 bp. All records from type C are based on previous analyses (for details see [23], [26], [59]).

### Molecular identification of *Niphargus*

Taxonomy and systematic of the genus *Niphargus* is still highly disputed and not yet resolved. The genus is known for a high level of cryptic diversity, and we thus grounded our identification based on molecular methods and a phylogenetic analysis. Samples with more than one individual per site were sequenced for two nuclear markers (partial 28S rRNA gene (28S) and histone 3 gene (H3)) that were already used in previous studies of Niphargidae [60]–[63]. Except in one case, samples containing only a single individual were not sequenced.

Genomic DNA was extracted using the GenElute Mammalian Genomic DNA Miniprep Kit (Sigma-Aldrich), following the Mammalian tissue preparation protocol. A fragment of 28S gene was amplified using primers from Verovnik et al. [64] (primer 5'- CAAGTACCGTGAGGGAAAGTT-3') and Zakšek et al. [65] (primer 5'-AGGGAAACTTCGGAGGGAACC-3'). The H3 gene was amplified using primers H3NF and H3NR from Colgan et al. [66]. PCR cycler settings are described in Fišer et al. [61]. PCR products were purified using the enzymes Exonuclease 1 and Alkaline phospathase (both Fermentas). Incubation consisted of two steps: 37°C for 45 min and 80°C for 15 min. PCR amplification primers were also used for sequencing. Contings were assembled and edited in Geneious 5.5.6. (Biomatters). Accession numbers for all sequences uploaded to GenBank are [will be provided upon acceptance of the manuscript].

### Analysis

For all taxa except the genus *Niphargus* we used accepted taxonomic and phylogenetic classifications [15], [19]–[21], [56]. For *Niphargus*, we first compared similarity of sequences with available comparative sequences from GenBank and unpublished sequences in our database (see http://niphargus.info/references/). In order to establish the taxonomic position of the Swiss *Niphargus* sequences, we performed a Bayesian analysis using concatenated dataset of two genes together with available sequences from GenBank [60], [62], [63], [67], [68].

All H3 gene sequences were of equal length (331 bp) and were unambiguously aligned using a simple algorithm (Geneious Alignment). The 28S rDNA sequences were highly variable in their length (761-904 bp) and were aligned in MAFFT ver. 6 [69] using the E-INS-i option for sequences with multiple conserved domains and long gaps. The optimal substitution model for each alignment was selected according to the Akaike information criterion in JMODELTEST 0.1.1. A GTR model of nucleotide substitution was selected for both genes, with gamma distributed rate heterogeneity for 28S and gamma distributed rate heterogeneity with a significant proportion of invariable sites for H3. Both alignments were concatenated and then analyzed in MRBAYES 3.2 [70] as two partitions. Two simultaneous runs with four chains each were run for five million generations, sampled every 100th generation. After discarding the first 25% of the sampled trees, the final topologies were constructed according to the 50% majority rule. Species identity was assigned on a basis of monophyly. We acknowledge that for identifying all potential cryptic species, a combination of further genetic markers may be recommended and that our approach is rather conservative and may not resolve all possible cryptic species within *Niphargus*. However, a complete phylogeny based on several genes is beyond the focus of this work and may also require more samples.

We compared the number of amphipod taxa (family, genus and species level) with the diversity found in neighboring countries as well as Slovenia, from which the amphipod fauna is well-known. We compiled information on amphipod diversity from Fauna Europaea (http://www.faunaeur.org/) as well as from the relevant literature (including [7,15,24,56,71–74). We used a thin plate spline surface to irregularly spaced data in order to predict species diversity patterns across Switzerland (function *Tps* in the R-package *fields* [75]), whereby the smoothing parameter is chosen by generalized cross-validation using default settings given by Nychka et al. [75]. When not mentioned differently, all statistical analyses were done with R version 3.0.1 [76].

## Results and Discussion

### Species diversity

We found a total of 29 different amphipod species in Switzerland, representing eight different genera (Fig. 1; table 2). 16 of these species (comprising three genera) are native to Switzerland, while 13 species are non-native, including five non-native genera. The herewith reported diversity of amphipods is much higher than what was previously published from Switzerland. For example, we find 100–140% higher diversity at the family, genera and species level compared to what is reported for Switzerland in Fauna Europaea [77] (Tables 1 and 2). However, we also note that several amphipod families (e.g., Ingolfiellidae, Bogiellidae, Hadziidae) found in neighboring countries (Table 1, [7], [8], [24], [71]) are not present in Switzerland, and thus the diversity in neighboring countries is generally higher. The lack of some major amphipod lineages may be due to the almost complete glaciation of Switzerland during the ice ages, as well as due to the lack of brackish water bodies, from which some species can invade freshwater systems.

**Table 2:** Checklist of all amphipods (class Crustacea, order Amphipoda) hitherto known from Switzerland, as well as tentative year of arrival for the non-native species.

Suprafamily	Family	Genus	Species	first record	comment
Talitroidea	Talitridae	*Orchestia* Leach, 1814	*Orchestia cavimana* Heller, 1865	2013	1
Crangonyctoidea	Crangonyctidae	*Crangonyx* Bate, 1859	*Crangonyx pseudogracilis* Bousfield, 1958	2007	
		*Synurella* Wrzesniowski, 1877	*Synurella ambulans* (F. Müller, 1846)	2001	
	Niphargidae	*Niphargus* Schiödte, 1849	*Niphargus auerbachi* Schellenberg, 1934	native	
			*Niphargus caspary* Pratz, 1866	native	2
			*Niphargus forelii* Humbert, 1877	native	3
			*Niphargus puteanus* Koch, 1836	native	
			*Niphargus rhenorhodanensis* Schellenberg, 1937	native	4
			*Niphargus setiferus* Schellenberg, 1937	native	
			*Niphargus thienemanni* Schellenberg, 1934	native	5
			*Niphargus thuringius* Schellenberg, 1934	native	
			*Niphargus virei* Chevreux, 1896	native	6
Gammaroidea	Gammaridae	*Gammarus* Fabricius, 1775	*Gammarus fossarum* Koch, 1835; Type A	native	7
			*Gammarus fossarum* Koch, 1835; Type B	native	7
			*Gammarus fossarum* Koch, 1835; Type C	native	7
			*Gammarus wautieri* A. L. Roux, 1967	native	8
			*Gammarus lacustris* Sars, 1863	native	
			*Gammarus pulex* (Linnaeus, 1758)	native	
			*Gammarus roeseli* Gervais, 1835	∼1850	
			*Gammarus tigrinus* Sexton, 1939	1990	
		*Echinogammarus* Stebbing, 1899	*Echinogammarus stammeri* S. Karaman, 1931	native	
			*Echinogammarus berilloni* (Catta, 1878)	∼1900	
			*Echinogammarus ischnus* Stebbing, 1899	mid-1990s	
			*Echinogammarus trichiatus* (Martynov, 1932	2004	
		*Dikerogammarus* Stebbing, 1899	*Dikerogammarus haemobaphes* (Eichwald, 1841)	∼1990	9
			*Dikerogammarus villosus* (Sovinskij, 1894)	late 1990s	
Corophioidea	Corophiidae	*Chelicorophium* Bousfield & Hoover, 1997	*Chelicorophium curvispinum* (G. O. Sars, 1895)	∼1980	
			*Chelicorophium robustum* (G. O. Sars, 1895)	2011	
			*Chelicorophium sowinskyi* (Martinov, 1924)	2011	

1 Ketmaier & De Matthaeis 2010 show that the continental European population is an undescribed but different species from the nominal species described from Cyprus, and will likely be given a different name. Ruffo et al. 2014 described it under the name “*Cryptorchestia garbinii*” as a new species based on specimens collected near lake Garda. For reasons of consistency and continuity, and with taxonomic work still ongoing, we use the name *Orchestia cavimana ( = Cryptorchestia cavimana* after Ruffo et al. 2014), but point out that the specimen reported might fall under what is now described as *Cryptorchestia garbinii*.

2 Probably comprises more than one species. Molecular analyses are needed to clarify taxonomic structure of the complex.

3 Populations in the type locality (Lake Geneva) possibly extinct.

4 A complex of at least six species (Lefébure et al. 2007), of which three are found in Switzerland.

5 A species closely related to *N. fontanus*, molecular analyses needed to clarify taxonomic structure of the complex.

6 Species complex with three morphologically similar species (Lefébure et al. 2006), of which one is found in Switzerland.

7 *G. fossarum* is a species complex with at least three species in Switzerland, called until formal description type A, B and C (Westram et al. 2011, 2013, Müller 1998, Weiss et al. 2013).

8 Karaman & Pinkster 1977 report it from the Jura mountains, and show a range-map extending into the Swiss Jura, but no specimens could be retrieved. Based on the species' distribution it is likely to occur in Switzerland (if it is not part of the *G. fossarum* complex) and its locality has been estimated from the map.

9 This species has likely been replaced by *D. villosus* and transient populations were found in Switzerland only for a few years.

Of the non-native species, one has been recorded in Switzerland around 1850 (*Gammarus roeseli*), while most others have been recorded in Switzerland for the first time over the last 30 years, which we interpret as a recent arrival. *Orchestia cavimana* is here reported for the first time for Switzerland (for a recent discussion of the taxonomic status of this species in northern Italy, see [78] and the footnote in Table 2). We found one individual in Lago di Lugano near Melide (Ticino, 45° 57′ 10.5″ N, 8° 57′ 10.9″ E) on July 2 2013. This species has its native range in south-eastern Europe [79], and has been found previously in the Po-region in Northern Italy [56]. Individuals from Lake Garda have recently been described as a distinct species, called *Cryptorchestia garbinii* [78]. For consistency, and lack of morphological differentiations that allow a clear assignment of the individual collected to either of these two taxa, we refer to it as *O. cavimana*. Furthermore, *O. cavimana* has also been found in the river Rhine in Southern Germany [34], but to our knowledge has not yet been confirmed from the Swiss part of the river Rhine. The most diverse amphipod genera within Switzerland are *Niphargus* (nine species) and *Gammarus* (eight species). Based on literature data (e.g., [22]), both of these genera very likely include further, overlooked cryptic species (Table 2). Such cryptic species are especially expected within the complexes of *G. fossarum*, *N. caspary*, *N. rhenorhodanensis*, *N. thienemanni* and *N. virei*.

Besides these 29 species, three further species have been previously reported for Switzerland (*N. aquilex, N. stygius and N. tatrensis*, [40]–[42], [80]). However, these records are very likely misidentifications and relate to species that do not occur in Switzerland. *Niphargus aquilex* was described from Great Britain [81]. A recent analysis suggests it comprises of a set of unrelated species not known from Central Europe [63]. The name *N. stygius* was broadly used for many species between Italy and Romania [82]. All *N. stygius* subspecies analyzed so far proved to be good species, which are completely unrelated to the nominal species [60]. The nominal species has a rather restricted distributional range along Italian-Slovenian border [83]. Finally, *Niphargus tatrensis* is restricted to the Carpathians [71], and the identification of specimens from Switzerland is not plausible. We suggest not including these three species in the amphipod fauna of Switzerland.

### Distribution patterns

**Figure 3:**
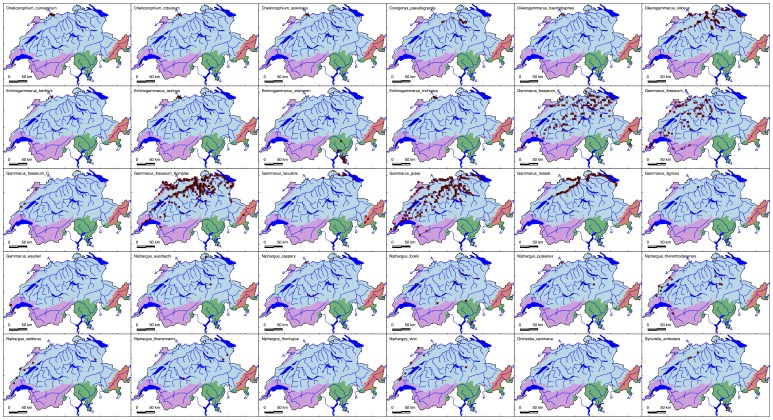
Distribution maps of all 29 amphipod species of Switzerland. Each panel gives the distribution of a species within Switzerland, in alphabetic order (see also Table 2). For *G. fossarum*, a map is given for the complex and the individual cryptic species respectively. Symbols show where the individuals were sampled: in lakes (circle), rivers and streams (square), or in the groundwater (diamond).

**Figure 4:**
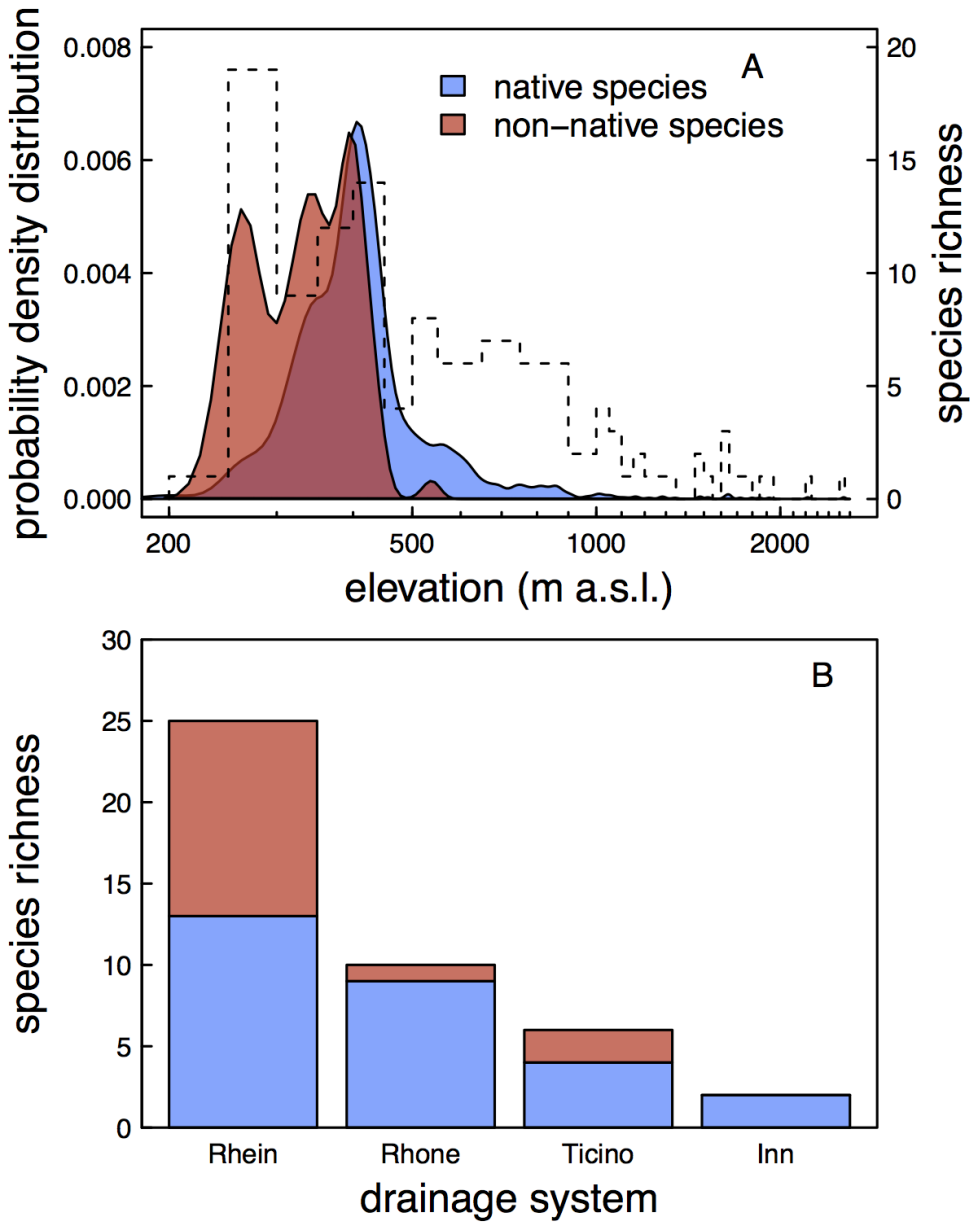
Occurrence of native and non-native amphipods relative to elevation and drainage basin. A) Occurrence of native and non-native amphipods relative to elevation. Probability density distributions are given for these two groups separately. The peaks of non-native amphipod occurrence at three elevations is linked to high sampling intensity at lakes in Ticino and River Rhine in Basel (elevation around 250 m), river Aare (elevation around 350 m) and Lake Constance (elevation 395 m). The dashed line gives the species richness at 50 m altitudinal bins. Note that the x-axis is on a log_10_-scale. B) Occurrence of native and non-native amphipod species across the four drainage basins in Switzerland.

The diversity of amphipods in Switzerland is highly uneven across the country as well as across different elevations (Figs. 2–4). The highest diversity is found in the High Rhine around Basel (47° 33′ 27″ N, 7° 35′ 33″ E), in lake Constance (47° 38′ 0″ N, 9° 22′ 0″ E) and in the river Aare (47° 36′ 22″ N, 8° 13′ 26″ E) before it drains into the river Rhine. In the 20×20 km square around the city of Basel, 14 species of amphipods were found (Fig. 2). This high diversity is due to a large number of non-native species (Fig. 3) found in the Upper Rhine [13], which are subsequently invading the High Rhine. The high diversity in the Upper Rhine and directly adjacent catchments is also supported by interpolated fits (Fig. 2C).

By far the highest number of records, but also the highest diversity of amphipods is found between 200 and 500 m a.s.l. (Fig. 4). However, amphipods could be found up to 2,540 m a.s.l. The non-native species are mostly found at lower elevations, and the altitudinal distribution of native and non-native amphipod species is significantly different (Kolmogorov-Smirnov test, D = 0.428, p<0.0001). This suggests that the non-native species originate from low-land areas (e.g., Ponto-Caspian area), and are actively invading the river system in the reverse flow direction. The distribution of records of non-native species shows three pronounced altitudinal peaks (Fig. 4A), reflecting both high sampling intensity but also high occurrence of non-native amphipods in the river Rhine around Basel (250 m a.s.l.), in the river Aare (around 350 m a.s.l.) and in Lake Constance (395 m a.s.l.). The three species with the highest elevation populations (and also with the largest altitudinal range in their distribution) were *N. forelii* (up to 2,540 m a.s.l.), *G. lacustris* (up to 1,918 m a.s.l.), and *G. fossarum A* (up to 1,850 m a.s.l.). Of the non-native species, *C. pseudogracilis* reached the highest altitudinal distribution at 538 m a.s.l.

**Figure 5:**
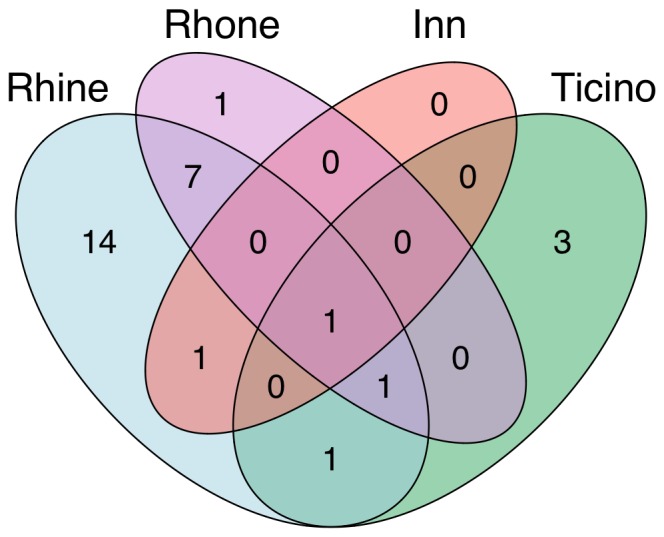
Venn diagram of amphipod co-occurrences. The Venn diagram is showing the number of co-occurring amphipod species across the four different drainage basins in Switzerland. The colors of the drainage basins follow Fig. 2.

The highest diversity in both native and non-native species was found in the river Rhine drainage (Fig. 4B). The other three drainage basins (river Rhone, river Ticino and river Inn) had lower numbers of native as well as non-native species, with the river Inn drainage basin being completely free of non-native amphipod species. We cannot exclude that part of the effect is due to some difference in sampling intensity among the drainage basins. However, the number of sites sampled (proportion of total sampling sites: 75% in Rhine, 17.5% in Rhone, 5% in Ticino, and 25% in Inn) is highly similar to the area these drainage basins cover (see Methods). Thus, we are confident that the sampling intensity between drainage basins is relatively similar, while there is some heterogeneity in the spatial location within drainage basins (see Fig. 2A). In the future, new methods such as the use of environmental DNA (eDNA) may allow to get an even better monitoring coverage [84], [85]. There are multiple mutually non-exclusive explanations for the difference in species composition between drainage basins. First, the river Rhine drainage basin is by far the largest drainage basin, and therefore a higher number of species is expected [86]. Second, the altitudinal range of the drainage basins differ, such that the river Rhine and river Ticino basin reach to the lowest altitudinal levels (246 and 238 m a.s.l respectively), allowing the potential invasion of typical lowland species. A third explanation would be the existence of northern refugia (see also [9]), and finally a predominant invasion from northern drainage basins (almost exclusively through the connection to the Ponto-Caspian area through channels between the Rhine-Danube system [13]). In analogy, the set of amphipod species found in the Ticino basin is almost completely different from all other drainage basins (Fig. 5), suggesting that all of them invaded Switzerland from Northern Italy in historic or recent times [56]. It is noteworthy that no non-native amphipod species has reached the Rhone river drainage basin from Southern France. The only non-native species in the Rhone drainage basin (*D. villosus*) is most likely the result of a secondary translocation from river/lake systems in the Rhine drainage basin [32], [87].

Only one amphipod species (*Gammarus fossarum* A) was found in all four drainage basins, and most of the others were restricted to one or two of the four drainage basins (Fig. 5). This indicates that processes that create and maintain species (e.g., local speciation, or persistence in refugia) as well as invasion processes unfold differently and separately in individual drainage basins. Most of the co-occurrences were found between the Rhine and the Rhone drainage basin. However, these, as well as all other co-occurrences across drainage basins, may need further investigation. Recent work on the *Gammarus fossarum* complex and on *Niphargus* shows that cryptic diversity is high across Europe, with many overlooked species [22], [60]. As the populations in Switzerland strongly differ in neutral population genetic markers [23], [26], we postulate that several of the existing “species” found across drainage basins may split up into different species. This has important consequences, as *Gammarus* is a model organism in ecotoxicology and commonly used as a bioindicator [16], [25], [88]: The largely overlapping distribution of at least three to eight *Gammarus* species in Switzerland (Fig. 3) highlights on the one hand the need for proper identification of these species and on the other hand calls for caution when individuals collected from natural populations are used for ecotoxicological tests. Translocations of individuals should be avoided due to risk of potential loss of endemic lineages and species, as observed in other freshwater organisms in (sub)alpine systems [10].

### Discussion of individual species other than the genus *Niphargus*

Of all amphipod records from Switzerland, species of the genus *Gammarus* were most common ones, both with respect to numbers of populations as well as local abundances (number of individuals, data not shown). Especially *G. fossarum* A, *G. fossarum* B, and *G. pulex* are widely distributed in the Rhine drainage basin. Interestingly, they are much less common in the other drainage basins, even though they are reported from all of them.

Of the non-native species, only *D. villosus, G. roeseli* and to some degree *C. pseudogracilis* have reached a wider distribution in Switzerland (Fig. 3). This is in strong contrast to the high to very high dominance of non-native species at a few sites [28], especially in the Upper and High Rhine (Fig. 2). While these non-native species have gained a lot of attention [28], [29], [33], [34], [43], their actual distribution in Switzerland is rather restricted to large rivers and lakes. It is unclear how much they are still spreading and if they would also be able to colonize most of the smaller tributaries and water bodies at higher elevations, as evidence of such dynamics is lacking. As many of the non-native species are originating from lowland habitats, and have mostly been found in larger water bodies, it is unlikely that they would colonize the majority of small headwater reaches in Switzerland. Furthermore, some of these non-native species (all members of the family Corophiidae) are filter feeders, and thus depend on sufficient amounts of suspended particles. It is possible that their successful establishment depends or indicates changes in water quality or in the amount of suspended organic particles.

### Discussion of the genus *Niphargus*

**Figure 6:**
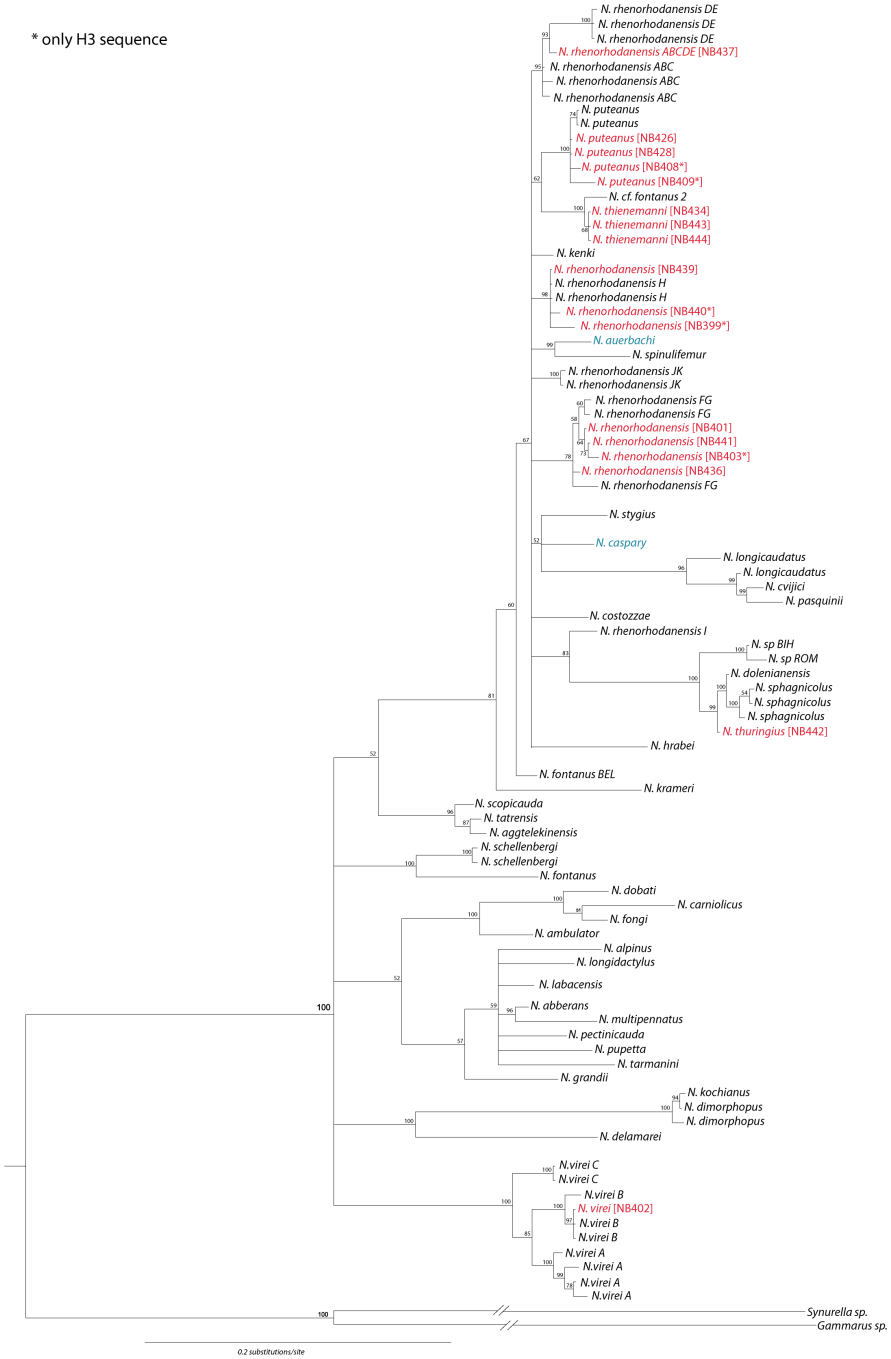
Bayesian phylogenetic tree of *Niphargus*. Samples from this study are in red, species occurring in Switzerland, but not sequenced within this project, are in blue. Numbers above nodes indicate posterior probabilities. Asterisk denotes specimens in which sequencing of 28S gene failed.

In total, we report the occurrence of at least nine species of *Niphargus* in Switzerland. The phylogenetic and geographic position of the nine *Niphargus* species collected in Switzerland shows that they are only distantly related to each other and have clear geographic affiliations to species outside Switzerland (Fig. 6). Members of four phyletic lineages (*Niphargus virei* and *N. rhenorhodanensis* complex) belong to lineages distributed mainly west of Switzerland. Three lineages (*N. thienemanni, N. puteanus*, *N. caspary*) are distributed predominantly north and north-east of the Alps, whereas *N. thuringius* belongs to a south-eastern lineage. Several of these species contain cryptic species, which have not yet been formally described. As this genus has been receiving little attention so far in Switzerland, we in the following discuss the status and possible cryptic species complexes for each species of *Niphargus* found in Switzerland.

***Niphargus auerbachi*** **.** This species is described from Switzerland (Schaffhausen, Schellenberg 1934) and reaches Southern Germany [89].

***Niphargus caspary*** **.** This species is broadly distributed across France, Switzerland, Germany, Austria, Hungary, Serbia and Romania. Considering the low dispersal abilities of subterranean species [24], it is unlikely that it is a single species across the whole range. Moreover, the species is morphologically variable. We reviewed a single sample, unfortunately too old to obtain a sample of intact DNA.

***Niphargus forelii*** is a species described from Lake Geneva, and reported from many other locations in Switzerland and deep alpine lakes from Italy and Germany [90]. An unsolved question is whether population from Lake Constance and other places in Switzerland and populations from Lake Geneva and surroundings belong to the same species.

***Niphargus puteanus*** **.** This species was originally described from Regensburg (Germany) and molecular data are available from individuals collected in Regensburg (type locality, [91]) and Tübingen (unpublished data). The samples from Switzerland are morphologically and molecularly almost identical to those from Regensburg.

***Niphargus rhenorhodanensis*** **.** Recently it has been found that this is not a single species, but a complex of at least six species [68], which do not even form a monophylum [63]. In 13 of our samples we found individuals belonging to the *N. rhenorhodanensis* complex. We successfully sequenced at least one sequence in eight out of fourteen individuals, and our data suggest that at least three species (lineage ABCDE, one sample; lineage FG, three samples; lineage H, four samples; lineage names according to Lefébure et al. [68]) of the complex live in Switzerland. No morphological revision exists and species identity can be assured by molecular markers only.

***Niphargus setiferus*** **.** The species was described from the French Jura mountains and reported from Switzerland by Strinati [42].

***Niphargus thienemanni*** **.** This species was identified in nine samples. It turned out to be molecularly closely similar to *N.* cf. *fontanus* 2 from Southern Germany [63], a sister species of *N. fontanus* from Great Britain [63], [92]. Most samples from above 1,000 meters contained only this species, a pattern already noted by Schellenberg [93].

***Niphargus thuringius*** **.** Described from Locarno, and primary considered as member of the *N. longicaudatus* species complex, it is distributed mainly in Northern Italy (Piemonte, Lombardia and Brescia). Contrary to expectations, in our molecular analyses this species turned out not to be related to the *N. longicaudatus* complex, but belongs to another clade of species that are distributed across Northern Italy (*N. dolenianesis*), Slovenia (*N. sphagnicolus* and another undescribed species), Bosnia and Herzegovina and Romania (both undescribed species), possibly also Slovakia (unpublished personal observations).

***Niphargus virei*** **.** The species was found in a single sample from the Jura mountains (the Jura mountains are the type locality). Recently, it was shown that the name *N. virei* in fact covers three morphologically similar species [67].

The phyletic diversity of *Niphargus* relative to species diversity (6 major phylogenetic lineages with at least 9 species) is relatively high in Switzerland, and may reflect complex patterns of diversification and colonization within this genus (Figs. 3 and 6). For comparison, the much more intensely studied groundwater fauna of Slovenia (which is about half the size of Switzerland) harbors 42 species of *Niphargus* belonging to seven major phylogenetic lineages. The relative high ratio of lineage to species diversity found in Switzerland might be explained by historical effects: large parts of Switzerland were covered by ice during the Pleistocene, and the large scale distributional patterns of subterranean crustaceans may testify the devastating effects of glacial cover on the subterranean fauna. An important notion is that the ranges of all *Niphargus* species found in Switzerland extend to areas that were not covered by glaciers. Such a pattern might have been caused by mass extinctions during Pleistocene and subsequent recolonization of the emptied subterranean environment. The time for post-Pleistocene within-country speciation, which would increase the species to lineage ratio, has likely been too short. Furthermore, it is reasonable to expect that species that colonized empty areas are good dispersers. Such species may maintain gene flow between the populations, which counteracts allopatric speciation.

The only species that might have survived all Pleistocene episodes under ice-sheet is *N. thienemanni* (see also [68], [94]). Its current altitudinal distribution reaches from 690 to almost 1,640 meters above the sea, suggesting it might tolerate a broad range of temperatures. Molecular and physiological analyses, however, are needed to test whether it truly survived glaciation episodes in the Alps, or whether it is merely a successfully dispersing species.

## Conclusions

Amphipods are important for ecosystem processes and trophic dynamics in freshwater ecosystems and increasingly important for eco-monitoring and ecotoxicology. Still, accurate data on the occurrence and distribution of amphipods are only available for some European countries (Table 1). We provide the first conclusive overview of the amphipod fauna of Switzerland. We found not only a much higher diversity than previously known, but also a highly uneven distribution of species across spatial and altitudinal gradients. Switzerland contains potentially important refugia and boreo-alpine relict populations, and is prone to large-scale invasions of amphipods from different parts of Europe.
